# Melanoma brain colonization involves the emergence of a brain-adaptive phenotype

**DOI:** 10.18632/oncoscience.11

**Published:** 2014-01-10

**Authors:** Vigdis Nygaard, Lina Prasmickaite, Kotryna Vasiliauskaite, Trevor Clancy, Eivind Hovig

**Affiliations:** ^1^ Department of Tumor Biology, Institute for Cancer Research, The Norwegian Radium Hospital, Oslo University Hospital, Oslo, 0310, Norway.

**Keywords:** brain metastasis, melanoma plasticity, glutamate signaling

## Abstract

The brain offers a unique microenvironment that plays an important role in the establishment and progression of metastasis. However, the molecular determinants that promote development of melanoma brain metastases are largely unknown. Utilizing two species of immune-compromised animals, with *in vivo* cultivated metastatic tissues along with their corresponding host tissues in a metastasis model, we here identify molecular events associated with melanoma brain metastases. We find that the transcriptional changes in the melanoma cells, as induced by the brain-microenvironment in both host species, reveal the opportunistic nature of melanoma in this biological context in rewiring the molecular framework of key molecular players with their associated biological processes. Specifically, we identify the existence of a neuron-like melanoma phenotype, which includes synaptic characteristics and a neurotransmission-like circuit involving glutamate. Regulation of gene transcription and neuron-like plasticity by Ca^2+^-dependent signaling appear to occur through glutamate receptor activation. The brain-adaptive phenotype was found as more prominent in the early metastatic growth phases compared to a later phase, emphasizing a temporal requirement of critical events in the successful colonization of the brain. Analysis of the host tissue uncovered a cooperative inflammatory microenvironment formed by activated host cells that permitted melanoma growth at the expense of the host organism. Combined experimental and computational approaches clearly highlighted genes and signaling pathways being shared with neurodegenerative diseases. Importantly, the identification of essential molecular networks that operate to promote the brain-adaptive phenotype is of clinical relevance, as they represent leads to urgently needed therapeutic targets.

## INTRODUCTION

Microenvironmental signal cues, to which melanoma cells themselves contribute, constantly shape the properties of melanoma through dynamic switching of transcriptional programs [1, 2]. Gene expression profiling studies have previously revealed the presence of two major signatures in melanoma, which distinguish two discrete states of differentiation; an invasive, slow-cycling mesenchymal-cell like phenotype, and a differentiated phenotype intrinsic to melanoma that is highly proliferative [1]. The expression of the invasive signature, driven by Wnt-signaling and TGFβ sensitive genes, has been found to be inversely correlated with the proliferative signature, driven by the MITF transcription factor. “Phenotype-switching” refers to the dynamic and reversible transition between the two phenotypes [3]. The invasive subtype resembles the phenotype of neuron progenitors during embryogenesis [4]. Melanomas and neurons share a common embryonal origin (neural crest derived), and the revival of a neuron progenitor phenotype may explain a “homing” phenomenon, and the frequent involvement of CNS metastasis in melanoma [5]. The brain offers a unique microenvironment that plays an important role in the establishment and progression of metastasis, though the molecular mechanisms of brain metastasis have not yet been clarified. However, the molecular factors now reported to be implicated include VEGF-A [6], STAT3 [7], p75 and neurotrophins [8, 9], transferrin [10], TGFβ [11] and endothelin receptor B (EDNRB) [12].

Injection of human tumor cells in immune-compromised animals offer a valuable model to collect *in vivo* cultivated metastatic material, thereby maintaining an environment closer to the clinical situation. Comparisons of metastatic specimens from various organs descending from the same parental cell line implicate organ-specific genes in organ-specific metastasis [13]. Further, the focus on the altered state and contribution of the host microenvironment has been neglected in many studies. The host microenvironment varies by organ, and the brain offers a unique milieu, which is also subjected to cellular and molecular changes following onset of disease. The activation of glial cells, such as astrocytes and microglial cells, are widely observed in CNS-related diseases, including brain metastases [14]. When activated, these cells represent sources of trophic and paracrine/autocrine factors that affect signaling cascades and the invasion, survival and growth properties of the incoming tumor cell [15].

In the current study, we applied different experimental metastatic melanoma models to generate effects both in metastatic tissues and in the corresponding host tissues *in vivo*, in order to investigate mediators of melanoma brain metastasis. Microarrays were used to analyze gene expression patterns in different tumor and host settings. Of significance, we identified a brain-adaptive phenotype that emerged in early/intermediate growth phase that encompassed the brain signature where glutamate signaling and Ca^2+^–dependent effectors played a central role, forming a top-down signaling pathway associated with neuron-like functions. The brain-adaptive phenotype offers an attractive melanoma brain metastasis target, as it may represent a cell population critical for brain colonization and equipped with signaling pathways that can escape current melanoma therapy.

## RESULTS

### *In vitro* cultured melanoma cell lines, Melmet 1 and Melmet 5, displayed distinct phenotypes of invasion and proliferation

The melanoma cell lines, Melmet 1 (MM1) and Melmet 5 (MM5), displayed distinct cellular phenotypes according to proliferation- and motility-based *in vitro* assays (Supporting Information Fig. S1*A*-*B*). MM1 is invasive with a lower degree of proliferation than the non-invading, rapidly dividing MM5 cell line. This assignment correlated with RNA expression levels of genes associated with either the invasive (*AXL*, *DKK3*, *WNT5B*) or proliferative (*MITF*, *TYR*, *MLANA*) phenotype specific signature, respectively (Supporting Information Fig. S1*C*).

### Organ-specific gene profiling in an experimental metastasis model revealed a brain-specific signature

**Figure 1:**
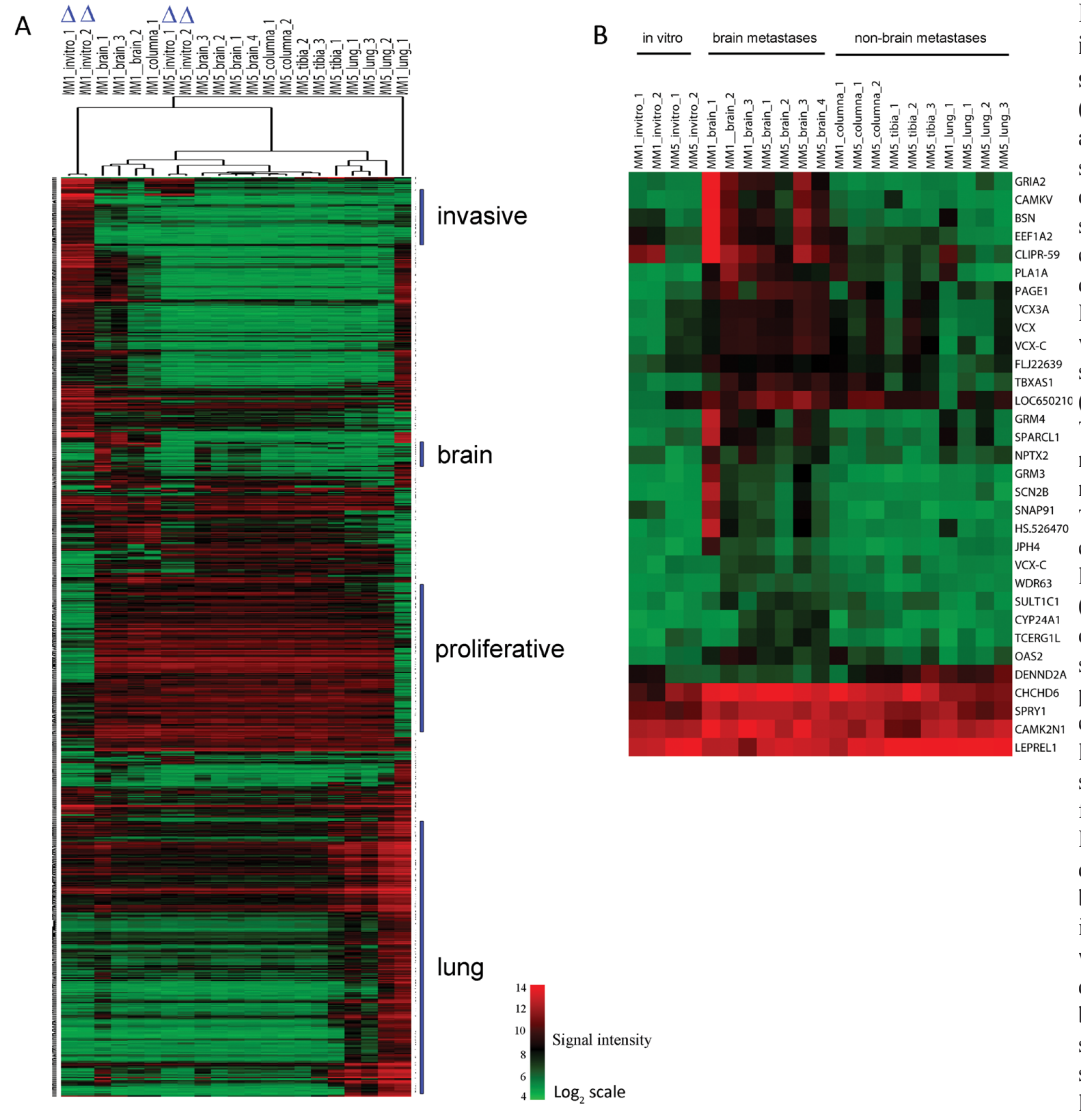
Signal intensity heatmaps of significant gene sets (*A*) Hierarchical clustering analysis of genes with significant variance across organ-specific metastasis specimens and *in vitro* cell cultures (marked with ∆) derived from MM1 and MM5 cell lines. Gene vectors with SD≥1 were selected for cluster analysis (844/20458 transcripts). The columns of the matrix represent samples and rows represent gene transcripts. The red/green color in each cell represents expression levels ranging from low (green) to high (red) levels on a log_2_-transformed scale. The invasive and proliferative signatures could not distinguish the cell lines *in vivo*. Organ-specific signatures were proposed for brain and lung. (*B*) Expression pattern of genes differentially expressed in brain metastasis. Signal intensity heatmap of genes with at least a 1.5-fold difference (FDR≤10%) between brain metastases samples vs. other *in vivo* sites (columna, tibia and lung) and *in vitro* samples.

Left ventricular injection of MM1 and MM5 (10^6^) cells respectively, into immune-suppressed rats caused development of metastasis in multiple organs, based on positive findings using immunomagnetic bead selection of melanoma cells in host tissues. To compare gene expression profiles from organ-specific metastatic lesions, we isolated metastatic melanoma cells from the brain, columna, tibia and lungs. MM1 was mainly represented by brain samples as the selected number of MM1 metastatic cells from organs other than brain was low. To explore the relationship between the 21 profiled specimens (Illumina HumanWG-6v3 beadarrays) representing *in vivo* and *in vitro* melanoma samples, we performed a hierarchical clustering analysis of normalized and log_2_-transformed gene expression data (Fig. 1*A*). The MM1 *in vivo* samples clustered closer to the MM5 *in vivo* samples than to their *in vitro* counterpart, and expressed genes representing the highly proliferative phenotype, such as the melanocytic markers *TYR* and *MLANA*, indicating a switch in phenotype of the MM1 progeny grown *in vivo.* A subset of genes showed an apparent organ-specific expression in either brain or lung. To determine the brain-specific gene expression, we assessed the number of genes differentially expressed in the brain metastasis samples (n=7), compared to the other *in vivo* (n= 10) and *in vitro* (n=4) specimens by Significance Analysis of Microarray (SAM). We found 32 genes to be up/down-regulated (FC=±1.5, FDR≤10%) in brain metastasis specimens (Supporting Information Dataset S1 and Fig. 1*B*). Neuron-specific functions were associated with up-regulated genes in this brain signature. Specifically, we found genes involved in neurotransmission (*GRIA2, GRM3, GRM4 and SCN2B*), neuron excitation (*CAMKV*, *JPH4*) and synaptic active zones (*BSN, SNAP91*). The canonical pathways which were most significantly associated with the up-regulated genes included glutamate receptor signaling (p-value= 2.7x10^−5^), neuropathic pain signaling (p-value=1.5x10^−4^) and synaptic long term potentiation (p-value=1.6x10^−4^).

### Growth phase-specific characteristics of melanoma brain metastasis; overcoming small samples limitations associated with early lesions

**Figure 2:**
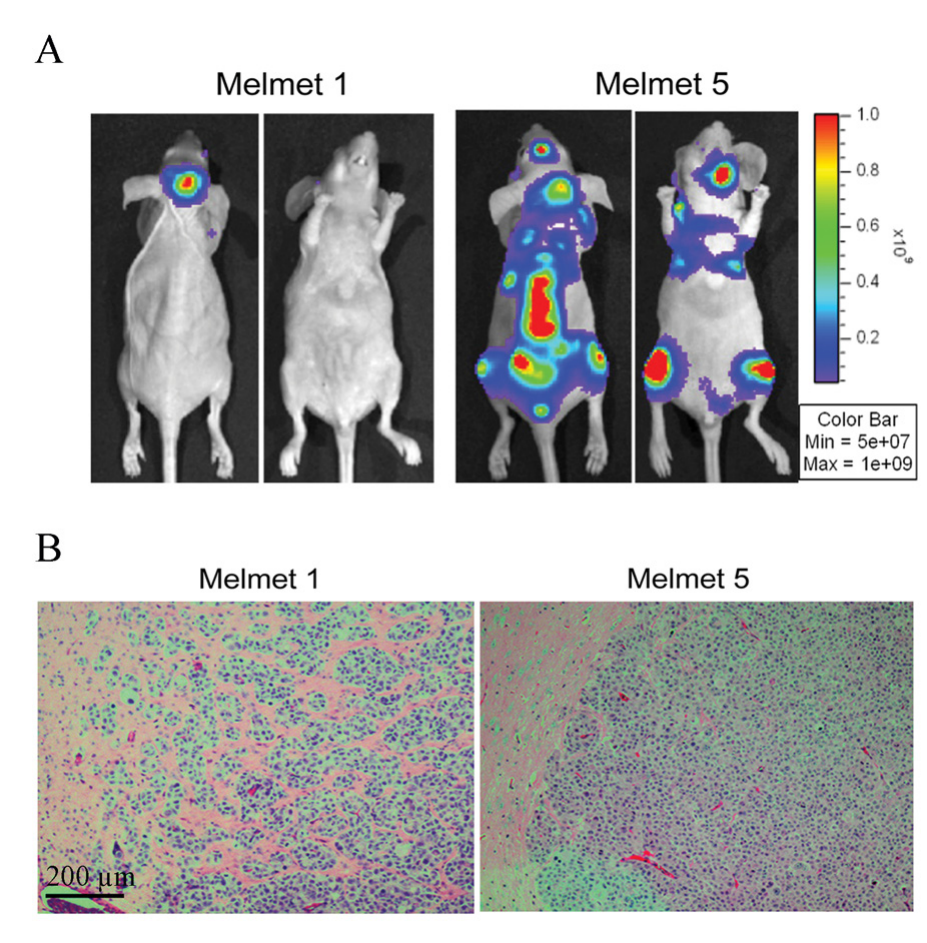
*In vivo* characterization of metastases development in nude mice injected with GFP-Luc tagged Melmet 1 (MM1) and Melmet 5 (MM5) cells (*A*) Representative bioluminescence images reflecting metastasis pattern characteristic for MM1 and MM5 cells. (*B*) Representative histology images of brain metastases detected by GFP staining reflecting invasive (MM1) and noninvasive (MM5) tumor growth pattern.

Having established the occurrence of phenotype-switching and the induction of a neuron-like phenotype specifically in brain metastasis in the model above, we tested in a second animal model whether we could further delineate the metastatic progression in the brain, by assaying melanoma cells at sequential time points after host injection. The use of a second host species would further verify the findings in the rat model. Through *in vivo* imaging, the cell line specific organ-growth preference was again observed, but now in a mouse model. MM5 metastasized to multiple organs, while MM1 preferentially grew in the brain (Fig. 2*A* and Supporting Information Fig. S2*A*). The brain metastases showed cell line specific growth rates and histological appearance (Supporting Information Fig. S2*B* and Fig. 2*B*). We assigned collected brain samples into three different growth phase categories (early, intermediate and late), based on *in vivo* imaging of brain metastasis growth in the respective host animals (Supporting Information Fig. S2*A*-*B*). Assaying tumor-specific gene expression in early stages of metastasis development is technically challenging, due to the small lesions. The procedure requires purification of tumor-specific RNA that complies with quantity and quality thresholds set in gene expression array protocols [16]. To maximize signals, we therefore hybridized an un-separated MM5/mouse sample representing early brain metastatic growth, onto a human-specific array (Illumina HumanHT-12 v4). We thus hybridized cRNA from one normal mouse brain specimen, one mixed-species sample, six metastatic melanoma cell samples purified from brain tissue in addition to parallels of the respective *in vitro* cell cultures injected into animals. Hierarchical clustering generated two main sample clusters that separated mouse-derived RNA, including the mixed-species sample, from human-derived RNA (Supporting Information Fig. S2*C*). The bead-detection P-value statistic defined by the GenomeStudio software (Illumina) was applied to solely quantify the extent of cross-species hybridization of mouse transcripts. For the pure mouse brain sample, 10.8% (4824/47324) of the transcripts passed the cut-off value (p< 0.05), compared to an average of 39.9% (18888/47324) in the human melanoma cell samples (Supporting Information Fig. S2*D*). In the mixed-species sample, 11.8% (5582/47324) of the transcripts passed the detection filter, indicating a contribution to the transcript number from the presence of human transcripts in the sample. We concluded that extracting informative data from the mixed-species depends on the hybridization design, as pure, relevant samples representing each of the species represented in the mixed sample were needed to deduce which genes were potentially of interest to the biological question.

### Network analysis of tumor transcripts altered by the brain microenvironment showed central roles for neuro-pathological associated genes

**Figure 3:**
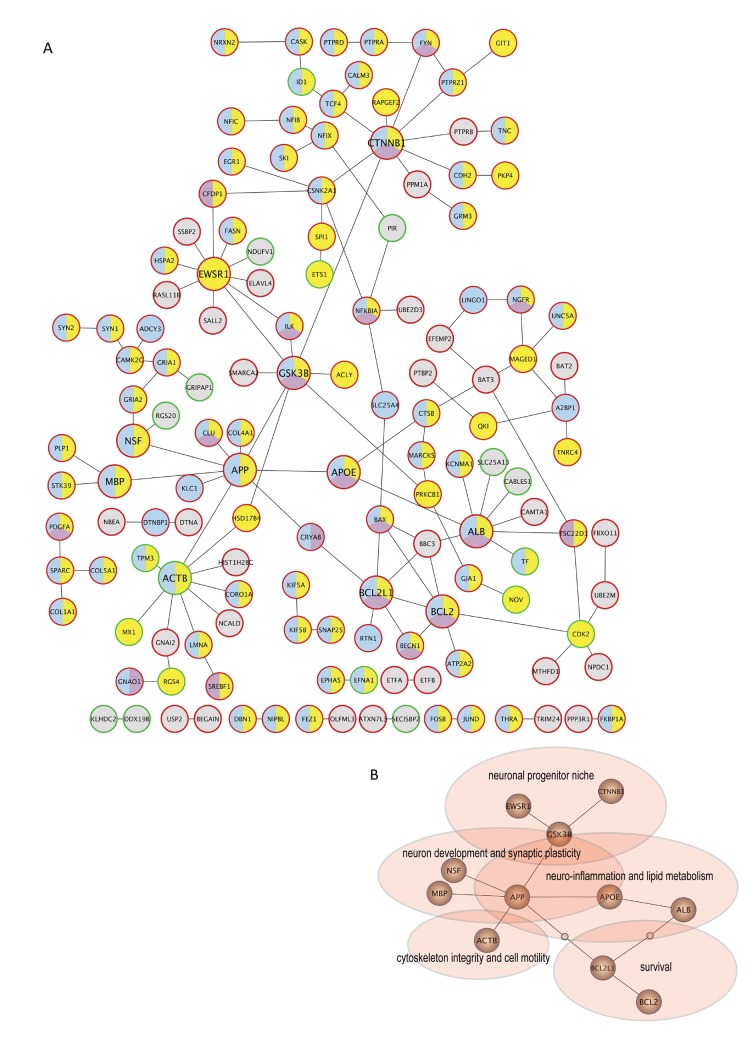
Protein-protein interaction network generated from genes with differential expression during metastatic growth in the brain (*A*) Protein-protein interaction (PPI) network based on differentially expressed genes between intermediate and late growth phase. The network was constructed from an integrated set of protein interaction database in combination with a gene ontology (GO) analysis). Lines indicate physical interaction between the proteins (circles). The outline of the circles indicate up (red)- or down (green)-regulated gene expression. The members of the network have been color-coded according to their association with the following biological functions; neurogenesis (blue), invasion (yellow) and survival (purple). (*B*) Reduced complexity network based on the central nodes in (*A*). The nodes shared common involvement in biological functions (spheres).

Using SAM analysis, we identified 697 differentially expressed genes (FC=±2) between intermediate (n=2) vs. late growth phase samples (n=4) (585 up-regulated/112 down-regulated, Supporting Information Dataset S2-S3). We generated a protein-protein interaction (PPI) network based on these 697 genes in combination with a gene ontology (GO) analysis in order to focus on central molecules and processes in our data set (Fig 3*A*). The PPI network was constructed from an integrated set of protein interaction databases whereby each interaction represented a known physical binding between two proteins. Six of the eleven central nodes were associated with all three investigated GO terms (neurogenesis, invasion and survival) indicating pleiotropic effects of these genes. The central nodes were re-drawn and key biological functions were distributed to best fit the central nodes and their associated node members (Fig. 3*B*). Notably, central genes (*APP, MBP*, and *APOE*) in the network are implicated in neuro-pathological disorders and metabolic disease, but have not previously been assigned significance in brain metastasis. The key biological functions including neuron development, neuro-inflammation, motility and survival, summarize the identified processes involved in melanoma adaptation and growth in the brain microenvironment based on the gene expression data obtained from our model and described below.

### Transcriptional dynamics during brain metastasis progression reflect an emerged brain-adaptive phenotype succeeded by highly proliferative melanocytic progeny

**Figure 4:**
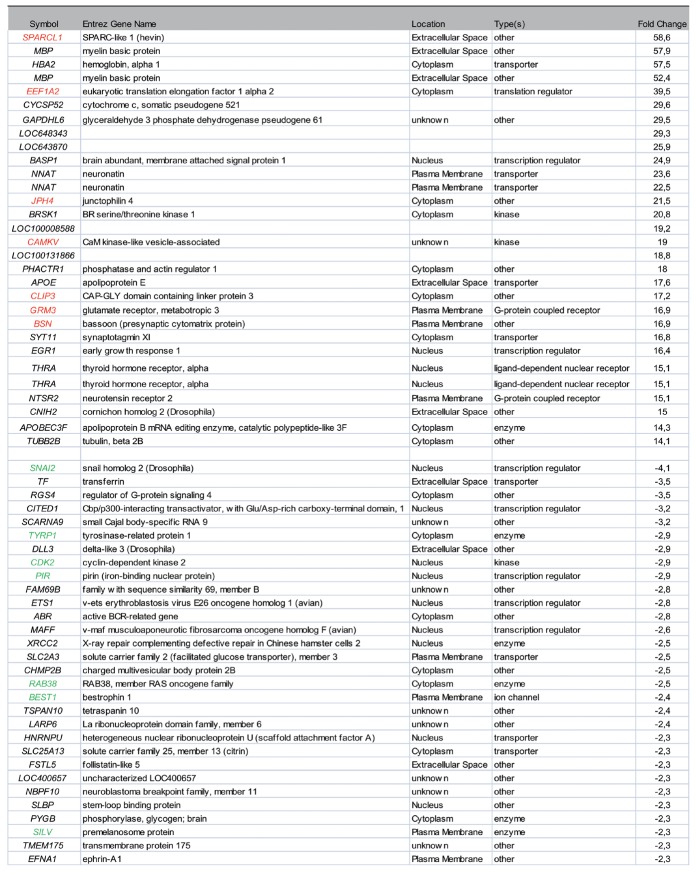
Top ranked up-/down-regulated genes in a comparative analysis of intermediate vs late growth phase melanoma brain metastasis in a mouse model. Genes marked in red overlapped with the brain-signature gene list obtained in the organ-specific profiling of metastatic melanoma using the same cell lines in a rat model. Genes marked in green indicate transcriptional targets of MITF.

**Figure 5:**
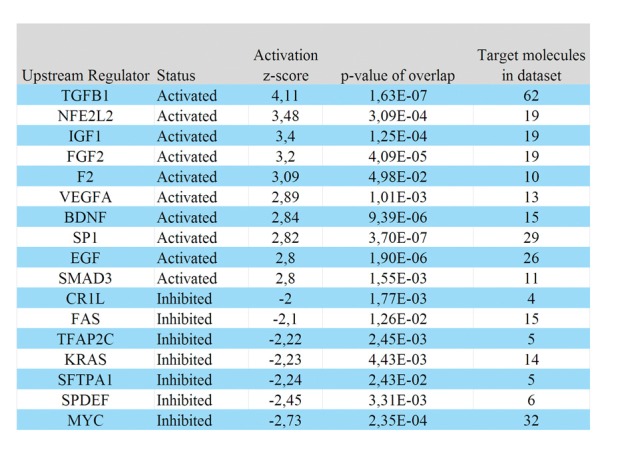
Top ranked activated/inhibited upstream regulators predicted for the given set of 477 up-regulated genes in the intermediate growth phase An activation z-score was calculated as a measure of concordance between the known affect of a transcription regulator has on each target gene compared with the observed gene expression changes. These relationships are associated with a direction of change that is either activating (z-score ≥ 2) or inhibiting (z-score ≤-2).

Among the top 30 genes up-regulated in intermediate growth phase, we found an overlap with 7 genes in the brain signature identified in the previous rat model data set (Fig. 4]. This overlap confirmed the involvement of these genes, and the acquisition of neuron-like characteristics in our model of melanoma metastasizing to the brain. The top 30 list encompassed further genes associated with the nervous system, including *SPARCL, MBP, BASP1, NNAT, BRSK1, PHACTR1, APOE* and *SYT11,* which are involved in processes such as normal brain development, myelination, neurite outgrowth and synaptic plasticity. The 585 up-regulated gene transcripts were mapped in the Ingenuity Knowledge (IPA) base where 477 identifiers were eligible for analysis of significantly associated biological functions and canonical pathways (Supporting Information Table S1). Top ranked functions included “neurotransmission”, “apoptosis” and “formation of plasma membrane projections”. Significantly associated canonical pathways included “Wnt/β-catenin signaling”, “PI3K/AKT signaling”, “axonal guidance signaling”, “CDK5 signaling”, “DHA signaling” and “glutamate receptor signaling”. The significance of the glutamate receptor signaling pathway was largely derived from the up-regulated receptors *GRIA1*, *GRIA2*, *GRM3* and *GRM4* (16.9- to 4-fold). We found a number of Ca^2+^-dependent effectors (Ca^2+^/calmodulin-dependent kinases, *CAMTA1*, *CAMTA2* and *CALM3*) that all were up-regulated. On the basis of the 477 identified genes, we queried known up-stream transcription regulators using IPA (Fig. 5, Supporting Information Table S1). Based on this analysis, *TGFβ* was the top predicted activated regulator, targeting 62 out of 477 genes. We noted activated status of *NFE2L2* (*NRF2*), a regulator of oxidative stress and metabolic reprogramming and the inhibited status of the tumor suppressors *FAS* and *SPDEF*. Of the 697 differentially regulated gene transcripts between the intermediate and late growth phases, 112 genes were down-regulated and represented genes with higher transcriptional activity in the late growth phase. Surprisingly, *SNAI2*, a transcription factor critical for the normal development of neural crest derived cells, had the greatest fold induction (4.1-fold) (Fig. 4]. However, *SNAI2* was recently reported to be co-expressed with *MITF* during melanoma progression [17]. We identified 6 additional genes among the top 30 up-regulated genes in the late growth phase as *MITF* targets (Fig. 4]. We noted that the MM1 d67 sample displayed low *MITF* expression, which explained why *MITF* itself was not in the list of differentially expressed genes. Significantly enriched biological functions associated with the mapped gene transcripts (97/112) included “synthesis of melanin”, “differentiation of melanocytes”, in addition to “cellular proliferation”, underlining the transition towards a differentiated/highly proliferative phenotype that predominates in the late growth phase (Supporting Information Table S2). TNF and MITF were assigned as activated upstream transcriptional regulators.

### Identification of molecular candidates orchestrating the metastatic niche and promoting the brain-adaptive phenotype

**Figure 6:**
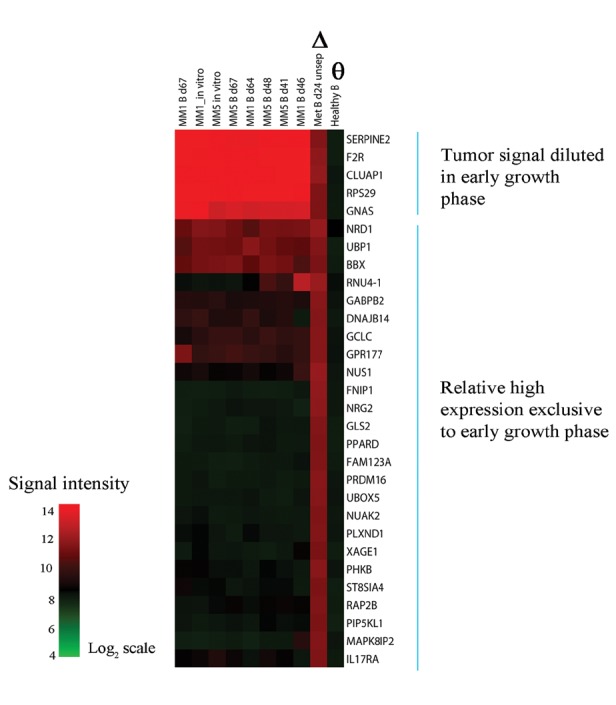
Signal intensity heatmap of the top 30 differentially expressed genes derived from a direct comparison between the mixed-species sample (early brain metastatic growth, marked with ∆) versus a normal mouse brain (marked with Ѳ) hybridized to a human-specific beadarray Columns represent samples and rows show gene expression levels. Comparison against pure tumor samples indicated that the expression level for the majority of the 30 genes was specific for the early growth sample while a minority could be interpreted as diluted tumor-specific signal.

From the gene expression data, we extracted genes with at least a 2-fold higher expression level in the mixed-species sample (MM5/mouse, representing early growth phase), compared to the normal mouse brain sample. The up-regulated genes (3848) were interpreted to belong to the categories of either tumor-specific genes, or cross-hybridizing genes in the diseased mouse brain with altered expression compared to the normal, healthy mouse brain (Supporting Information Dataset S4). Signal intensity alignment of the top 30 up-regulated genes was inspected across all samples so as to evaluate which genes could potentially be associated with early growth of melanoma in the brain (Fig. 6]. The top 5 gene rows appeared to be genes with a diluted tumor-specific signal, and thus not specific to either early growth phase or organ. The genes reported below, however, showed exclusive high expression in the mixed-species sample. The most differentially expressed gene, *NUS1* (*Nogo-B* receptor) (13.8-fold), is involved in angiogenesis and migration. Energy sensing and metabolism were related to the genes *GLS2, FNIP1*, *PHKB, PPARD, GCLC* and *NUAK2*. Neuregulin growth factor *NRG2* (12.3 fold), a ligand for the ERBB receptor family was also among the top 30 genes. The main results of significantly associated biological functions and canonical pathways for the mapped genes in the dataset (2412/3848) are reported in Supporting Information Table S3. Highly ranked annotated functions included processes such as “growth of neurites”, “metastasis of tumor” and “development of fetal membranes”. “EIF2 Signaling” and “Ephrin Receptor Signaling” were highly ranked in the list of significant canonical pathways reflecting stress, immune response and cell-cell interactions. The identification of RAF1 as a predicted activated upstream regulator was not surprising considering this analysis involved a comparison between neoplastic and normal tissue. The output from the upstream regulator analysis also included activated status for EGR2 and HNF4A. HNF4A, a transcription factor involved in lipid metabolism, possesses known regulatory functions towards 246 gene targets up-regulated in the early growth sample. Several microRNAs were identified as inhibited upstream regulators. DICER1, a key component in the miRNA processing machinery was also assigned to inhibited status, albeit not within the significance cut-off score. Down-regulated genes in the mixed-species sample were not pursued due to the likelihood of confounding factors. Briefly, a large number of down-regulated genes were associated with cell death and cellular metabolism. Pathway analysis showed associations to gliosis and astrocytosis.

To further characterize gene expression that could be particularly associated with the early growth phase of brain metastasis, we performed an unsupervised k-means clustering of the global gene expression in the whole data set (10 samples). The data set was distributed into 20 gene clusters that captured the main variance in the data. One cluster showed a distinct signal peak pattern exclusive to the mixed-species sample (Supporting Information Fig. S3). Growth factors such as *NRG2*, *PTN*, *VEGFA*, *PDGFD*, as well as the neurotrophic factor *GDNF* and ECM remodeling factors, including *MMP3*, *MMP16*, *MMP21*, *COL6A*, *COL8A*, *FBLN5*, *HAPLN2* and *SPON1,* were all associated with the cluster. The main results of significantly associated biological functions and canonical pathways for the mapped transcripts (1201/2135) in this cluster are reported in Supporting Information Table S4. Enriched functions included “translocation of lipid”, “muscular dystrophy” and ”regulation of l-glutamic acid”. Leptin and DHA signaling pathways were highly ranked, emphasizing a role for metabolic signaling in early metastatic growth. In a proteomic approach to identify factors associated with the early metastatic niche, we utilized a high density antibody array (RayBiotech) for screening the expression of 507 proteins. In a comparison between whole brain tissue lysates prepared from MM5 early metastatic growth phase brain (n=3) versus normal mouse brain (n=3), we found 30 differentially expressed proteins (FC=≥0.5) (Supporting Information Dataset S5). Several extrinsic factors previously reported to be associated with the tumor microenvironment were identified, including GCSF, THBS1, S100A8/A9, CCL2, TNF and TNFSF11. We noted the altered levels of NRG2 (and NRG3), consistent with up-regulated *NRG2* at the transcriptional level. The 6 predicted upstream regulators (IL1β, TNF, OSM, IL6, MYD88 and IL17A) assigned to the 30 proteins are all linked with neuro-inflammation and activated glial cells (18-20).

### Profiling of brain stroma revealed an immune responsive host microenvironment with altered host cell functions

Brain tissue analysis by flow cytometry showed recruitment and accumulation of inflammation associated myeloid cells in the metastatic brain (Supporting Information Fig. S4*A*-*B*). Using Illumina RatRef-12 beadarrays we profiled host brain stroma RNA (n=7) in order to capture altered gene expression due to melanoma invasion and growth. The stroma samples were representative of the microenvironment at a late growth phase. These profiles were compared to healthy rat brain (n=3). SAM analysis identified 45 differentially expressed genes (FC±2, FDR≤10%), including induction of components of the complement system (*C1qc*, *C1qa*, *C1qb*), cytokines (*Ccl3*, *Ccl4*), growth factors (*Grn*) and galectins (*Lgals1* and *Lgals3*) (Supplementary Dataset S6). Several of these genes are associated with the innate immune system which remains intact in our athymic host animals. We verified and complemented these findings in the mouse model, where the equivalent host brain stroma samples (late growth phase, n=3) were compared against normal mouse brain tissue (n=3) (Illumina MouseWG-6 v2) (Supporting Information Dataset S6, last column). In this mouse model, SAM analysis identified 769 differentially expressed transcripts (FC±2, FDR≤10%) between host brain stroma and normal brain tissue (433 up-regulated/335 down-regulated, Supporting Information Dataset S7). The top up-regulated gene in the surrounding stroma was identified as histone demethylase *Kdm5c* (alias *Jarid1c*) (52-fold). We found increased expression of *Ckmt1a (*24-fold), a creatine kinase involved in cellular energy homeostasis. *Spp1* (osteopontin) was highly up-regulated (22-fold), consistent with reports of significant overexpression of this gene in patient material and experimental metastasis models [21, 22]. We noted an overlap of up-regulated genes with an immune signature reported in a glioblastoma profiling study, including MHC class I and II genes, the complement factors *C1QA/B/C*, *FCGR3A*, *B2M*, *SOCS3*, *TLR2* and *CTSH* [23]. Activation of an immune response was reflected in the biological functions, canonical pathways and predicted upstream regulators significantly associated with the gene list (Supporting Information Table S5). Neuron-related functions were highly associated with the down-regulated genes in the host tissue, potentially reflecting a reduced number of functioning neurons (Supplementary Table S6). Further, the reduced levels of metabolically associated enzymes, kinases and ion channels indicated altered or dysfunctional host cells.

### Support for glutamate receptor signaling as a potential therapeutic target

**Figure 7:**
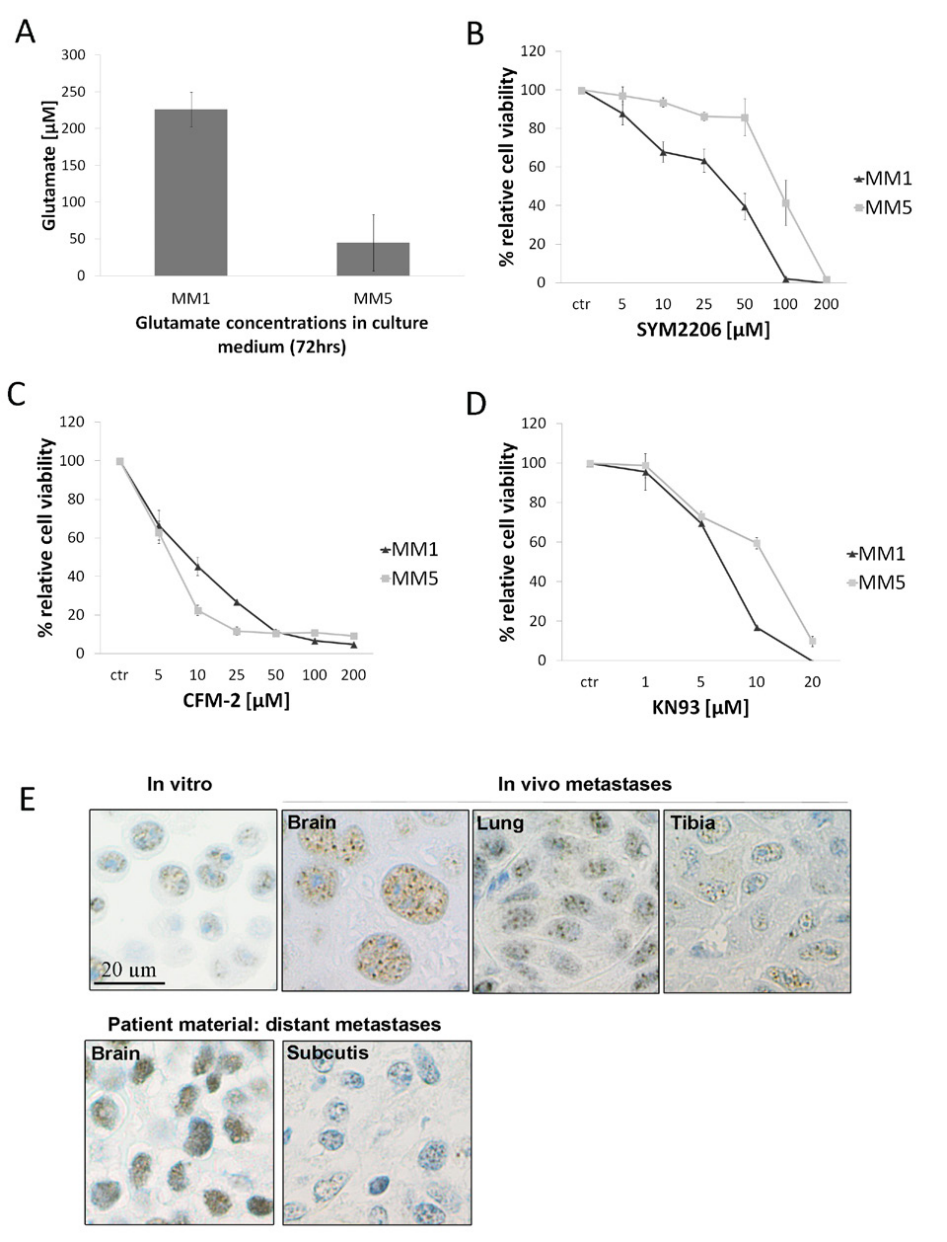
Glutamate receptor signaling and immunohistochemical validation of *in vitro* and *in vivo* (*A*) Glutamate concentration measured in medium after 72 hours of culture. Cells were plated in 96-well plates at 5x103 cells per well and 10 μl were collected (10% of media) (*B-C*) The AMPA antagonists SYM2206 and CFM-2 exert antiproliferative effects on both Melmet cell lines. Cells were exposed to vehicle control (DMSO) or 5-200 μM SYM2206 or CFM-2 for 72 hours, and viability was measured by means of the MTS assay. (*D*) The CaMKII inhibitor, KN93, decreased viability of both Melmet cell lines in the MTS assay. (*E*) (*Upper panel*) Representative H&E sections stained for the glutamate receptor, GRIA2 based on fixated MM5 cells from *in vitro* cultures and MM5 brain, lung and tibia metastases obtained from the experimental metastasis mouse model. The brain section showed stronger staining of GRIA2 with a nuclear localization. (*Lower panel*) The brain metastasis section from the patient from which the MM1 cell line was derived, also showed stronger nuclear staining of GRIA2 compared to the patient-matched subcutaneous lesion. See Supplementary Fig. S5C for additional MM1 stained sections.

Based on the involvement of glutamate receptors in the brain-signature, we investigated the presence of functional glutamate signaling in MM1 and MM5 *in vitro.* We first established that MM1 raised the level of extracellular glutamate in the culture medium six times more than MM5 after 72 hours (Fig. 7*A*). GRIA2 is a subunit of the AMPA glutamate receptor family (GRIA1-4), and with real time PCR analyses, we detected low and differential expression of the AMPA subunits in MM1 and MM5 (Supporting Information Fig. S5*A*). Blockade of AMPA receptors by means of the antagonists CFM-2 and SYM2206 suppressed cell viability of both cell lines (Fig. 7*B-C*). Glutamate receptor activation allows the influx of Ca^2+^ and activation of downstream Ca^2+^-dependent effectors. Both cell lines showed sensitivity to the calcium/calmodulin-dependent protein kinase II (CaMKII) inhibitor, KN93 (Fig. 7*D*). To verify the induction of neuronal gene expression in our metastatic melanoma cell lines when grown in a brain microenvironment, we cultured MM1 in brain-conditioned medium *in vitro*. The expression of *GRIA2*, *GRM4* and *BSN* increased in the presence of brain-conditioned medium relative to control medium (Supporting Information Fig. S5*B*). Immunohistochemical detection of GRIA2 was performed on metastatic sections from both the animal model and on patient material. In the brain, GRIA2 staining was detected solely in the nuclear compartment where the staining was stronger when compared to other metastatic sites (Fig. 7*E* and Supporting Information Fig. S5*C*). This was also observed in the metastatic tissue sections obtained from patient material from which the MM1 cell line was derived (Fig. 7*E*). Collectively, these data support a role for glutamate receptor signalling in cellular growth of MM1 and MM5 *in vitro*. The glutamate receptor signalling appears enhanced in the brain microenvironment, and thus represents a targeting opportunity.

## DISCUSSION

In this study, we revealed a number of molecular and cellular characteristics of melanoma brain metastasis by means of experimental metastasis modeling, drawing information from two melanoma cell lines with distinct phenotypes (invasive/proliferative) *in vitro*. Global gene expression analysis of the cell lines clearly showed that the environmental context was important in shaping the gene expression profiles. Although the analysis was based on a limited number of samples, the data was found to be consistent across two separate models in different host species. The interconnectivity of the differentially expressed genes allowed us to delineate the scenario of biological processes involved in melanoma brain colonization for further investigation. The data reflected an adaptation phase of melanoma cells to the brain microenvironment that required a specific phenotype (brain-adaptive) before a phenotype-switch was triggered, allowing expansion and production of the bulk tumor mass.

The brain-specific signature identified in melanoma cells that metastasized to the brain, showed that these cells acquired neuron-like characteristics. This signature showed a striking overlap with a recent report highlighting the significant transcript differences between xenografted breast cancer cells harvested from brain versus other mouse organs [24]. We identified the involvement of glutamate signaling in melanoma brain metastasis and showed that glutamate receptor inhibition reduced growth *in vitro*. These findings, suggest a therapeutic potential of targeting glutamate-induced signaling pathways. Dysregulation of glutamate signaling has been observed in several cancer types including melanoma. The metabotropic glutamate receptor 1 (*GRM1*) has been implicated in melanoma development [25]. Sequencing of melanoma samples identified the glutamate receptor subunit *GRIN2A* as the most frequently mutated gene [26]. Frequent mutations were also discovered in the *GRM3* metabotropic receptor gene [27]. The particular nuclear localization of GRIA2 that we found in brain metastasis from both animal model and patient material may define its signaling specificity in melanoma brain metastasis, which requires further investigation. Nuclear staining of GRIA2 has been reported for certain tumor cases, including melanoma, in The Human Protein Atlas database (http://www.proteinatlas.org/). In neurons, glutamate-induced Ca^2+^ influx affects the activity of Ca^2+^-dependent transcriptional regulators, such as CREB, EGR1, EGR2, NFAT and NF-κB, thereby regulating transcription of genes with important roles in survival, synaptic development and neuronal plasticity. The Ca^2+^–dependent transcription regulators mentioned above were predicted as activated in the upstream regulator analyses performed on datasets obtained from early/intermediate growth samples, indicating a functional Ca^2+^-dependent signaling system in metastatic melanoma in the brain. Enhanced influx of intracellular Ca^2+^ through increased expression of glutamate receptors requires mechanisms to avoid Ca^2+^-overload, which may cause cellular stress and trigger cell death. In our data collected from the intermediate growth phase samples, we found up-regulation of genes associated with gap junctions, which may allow increased diffusion of Ca^2+^ to neighboring astrocytes. Astrocytes have been described as recipients of excessive Ca^2+^ through gap junctions, thereby protecting the melanoma cells [28]. Blocking this astrocyte-dependent clearance service could therefore represent a potential target in melanoma brain metastasis. The key biological functions associated with the brain-adaptive phenotype also included energy sensing and lipid metabolism. Lipid signaling regulates a number of activities related to membrane structures, receptor signaling, cell metabolites and energy homeostasis. Further, melanoma cells endowed with a higher efficiency in utilizing the microenvironmental signals derived from the brain milieu would gain an advantage in producing brain metastasis. In our model, the MM1 cell line consistently produced stronger signals for the neuron-like genes. In addition, the preferred site of metastasis was the brain. These properties suggest that this cell line represents a very useful tool when focusing on molecular mediators of brain metastasis.

From an overview of altered gene expression in the host tissue, we postulate that glial cells were activated, while neurons were displaced. IL1β and TNFα, key drivers in neuro-inflammation were predicted to be activated upstream regulators of altered genes/proteins in host brain stroma. TNFα potentially represents a link between immune factors and glutamate receptor activation as glutamate has been shown to stimulate migration of microglial cells to the tumor [29]. The identified up-regulation of the histone demethylase *Kdm5c* transcription level requires further validation, but is an intriguing observation suggesting an epigenetic regulation of host stroma. To our knowledge, there are no reports of KDMs regulating stromal cell transcription in cancer, but epigenetic regulation by histone demethylases, including *KDM5C*, have been reported in neuro-pathological disorders [30].

Collectively, the data from tumor and host profiling provided evidence of favorable growth conditions set in a pro-inflammatory microenvironment created by the host cells, whereby we hypothesize that by hijacking normal neuron functions, melanoma cells enhanced interactions with activated glial cells that permitted melanoma survival and growth at the expense of the host organism. The development of a neural circuit, involving glutamate, glutamate receptor-operated Ca^2+^ channels, Ca^2+^ -dependent gene transcription, host glial cells and modulators fine tuning the system, defined essential properties of the brain-adaptive phenotype. Central molecular factors and pathways mediating melanoma brain metastasis and the brain-adaptive phenotype defined in this report are also central to other pathological diseases, in particular neurodegenerative and metabolic diseases (*e.g*. Alzheimer's and diabetes).Aberrant glutamate signaling is a recurring theme in several major neurological disorders and is also involved in brain metastasis as we demonstrate here. The PPI/GO-network reveals pathogenic convergence *i.e*. pleiotropic genes hold central positions in a PPI signaling network that affects several diseases. This suggests that these genes act as connectors between different functional pathways in the same PPI context as activated in different tissue settings. Similarly, the observed immune response with key inflammatory drivers found in this study, are also common to neuropathology and metabolic disorders. Investigations of the shared pathways may benefit the understanding of the underlying biological mechanisms, and may boost the search for effective targeting of brain metastases. An important extension of the results presented in this report will be to characterize the biological role of the significant gene expression patterns identified, and to evaluate potential therapeutic targets within these networks.

## METHODS

### Tissue source and cell cultures

The malignant melanoma cell lines Melmet 1 (MM1) and Melmet 5 (MM5) were established from melanoma patient subcutaneous and lymph node metastases, respectively, at the Norwegian Radium Hospital (Oslo University Hospital, Norway) as described previously [31]. Green fluorescent protein-luciferase (GFP-Luc)–labelled melanoma cells were generated by transducing the cells with lentivirus carrying a human ferritin promoter-driven GFP-Luc construct described previously [31, 32] (kindly provided by Dr. G.Merlino, NIH, MD). The cells were routinely cultured in RPMI 1640 medium (Lonza), supplemented with 10% fetal calf serum (FCS) (PAA) and 2 mM GlutaMAX (Gibco). Assessment of cell proliferation and motility *in vitro* is described in Supporting Information (SI).

### Experimental metastasis models *in vivo* and tissue preparation

All experiments using animals were approved by the National Animal Research Authority and were conducted according to the regulations of European Laboratory Animals Science Association. To generate experimental metastasis in rats, 200 μl of cell suspension containing 10^6^ cells from low passage cell cultures of MM1 (passage 8) and MM5 (passage 9) was injected into the left ventricle (l.v.) of Rowett nude rats (female, 4-5 weeks of age). Metastatic organs (brain, lung, tibia and columna) were collected and used for isolation of tumor and host stroma cell fractions. The equivalent organs were also collected from untreated control rats and processed in same fashion as described below. Organs were disaggregated and suspended in cold PBS/0.4% human serum albumin (HSA) (Octapharma). Lung samples were treated with ACK lysis buffer (Lonza) for 5 minutes (RT) to remove red blood cells and re-centrifuged. Melanoma-binding immunomagnetic beads were added to cell suspensions to separate positive tumor cells from negative stroma cells as described previously. The isolated tumor and stroma fractions were subjected to RNA isolation as described below.

To generate experimental metastases in mice, 200 μl of cell suspension containing 10^5^ GFP-Luc tagged MM1 and MM5 cells (passages under 25) were injected l.v. into athymic FOXN1 nude mice from Harlan (5-7 weeks of age). Metastatic growth was followed by repeated non-invasive bioluminescence imaging using the *in vivo* imaging system IVIS (Xenogen) following intraperitoneal injection of 200 μl D-luciferin (Biosynth). Images were analyzed by Living Image 2.5 software. The animals were sacrificed at different time-points post injection and brains harvested and processed as described above. The purity of the separated fractions was inspected by fluorescent microscope observing GFP^+^ tumor cells in the positive fraction and lack of GFP^+^ cells in the negative (stroma) fraction. In parallel, the harvested organs were fixed in 4% formalin and embedded in paraffin for immunohistochemistry. The presence/absence of metastases was confirmed by hematoxylin and eosin (H&E) staining performed following standard protocols.

### Flow cytometry

Approximate 1/10 of the total cell suspension prepared from the disintegrated metastatic or healthy brain as described above was re-suspended in 100 μl cold staining buffer (PBS containing 1mg/ml human γ-globulins (Sigma) pre-treated at 63°C for 20min) and incubated with the following antibodies: 1μl V450 anti-mouse CD11b (clone M1/70, BD Biosciences) and 0.5 μl eFluor 660 anti-mouse Gr-1 (clone RB6-8C5, eBiosciences) or 5 μl AlexaFluor 647 anti-mouse F4/80 (Cl:A3-1, AbD Serotec). Following 30 min incubation at 4°C, the cells were centrifuged, re-suspended in RPMI/2 % FCS and analysed by LSRII flow cytometer (Becton Dickinson) using BD FACSDiva™ software. Minimum 10,000 events from GFP-negative (stroma) cell population were collected for each sample. FlowJo 7.2.5 software was used to analyse the data.

### Collection of gene expression data

*In vitro* cell cultures and positive melanoma fractions applied in the rat model were isolated using Absolutely RNA Microprep Kit (Stratagene). Total RNA from negative host stroma fractions and all melanoma samples collected from the equivalent mouse model were isolated with TRI Reagent^®^ (Invitrogen). RNA was amplified from 200 ng (rat model samples)/500ng (mouse model samples) total RNA using the Illumina TotalPrep RNA amplification kit (Ambion Inc) following manufacturer's protocol. For each sample, 750 ng labeled cRNA was hybridized to one of the following Illumina beadchips; HumanWG-6 v3, Human HT-12 v4, RatRef 12 v1, and MouseWG-6 v2 (Illumina Inc). Hybridization and scanning were performed by the Norwegian Radium Hospital Microarray Core Facility according to Illumina protocols. Raw microarray data sets were quantile normalized and log_2_-transformed. Pre-processed microarray data are available through the EBI Array Express database, http://www.ebi.ac.uk/arrayexpress/ accession no. E-MTAB-2065. Detailed methods of data analyses and generation of the protein interaction network can be found in Supplementary Information (SI).

For qRT-PCR reactions, 1 μg total RNA was first reverse transcribed using qScript cDNA Sythesis Kit (Quanta Biosciences) according to manufacturer's protocol. Details of qRT-PCR reaction and primer sequences can be found in Supplementary Information (SI).

### Measurement of extracellular glutamate

Glutamate assay kit (BioVision) was used to measure the amount of glutamate released into the medium. Cell were plated in 96-well plates at 5x10^3^ cell per well in 100 μl medium. After 72 hours 10 ul of medium was collected for glutamate measurement according to manufacture's protocol.

### Cell viability assay

Cells were plated in 96-well plates at a density of 4x10^3^ (MM1) and 8x10^3^ (MM5). After 24 hours, the medium was removed and cells were exposed to serial dilutions of SYM2206, CFM-2, KN93, l-glutamate or AMPA, all purchased from Tocris Bioscience. Cell viability was assessed after 72 hours by using the CellTiter 96 AQueous cell proliferation assay kit (Promega).

### Effect of brain-conditioned medium on gene expression

The brains were collected from healthy mice or mice injected with MM5 cells at day 7 post-injection. The organs were cut into 1 mm^3^ fragments, re-suspended in serum-free DMEM/GlutaMAX medium (125 mg tissue/1 ml medium), transferred into cell culture flask and incubated at 37°C for 3 hrs under rotation. Subsequently, the supernatant was filtrated through 70 μm and 0.2 μm filters to obtain cell-free brain-conditioned medium. MM1 cells (3x10^4^ cells/well) were seeded out in a 12-well plate. The next day the culture medium was replaced by brain-conditioned medium diluted with DMEMat a ratio 1:4 and supplemented with 5% FCS. After incubation for 72hrs at 37°C, the cells were collected for RNA extraction and qRT-PCR analysis.

### Immunohistochemistry (IHC)

Paraffin-embedded (5μm) tissue sections were pre-treated for deparaffinization, rehydration and epitope retrieval using an automated PT-link system (Dako) and stained with polyclonal rabbit antibodies against: GRIA2 (Abcam ab52176, 1:200), CD11b (Abcam ab75476, 1:600) or monoclonal mouse antibody against Ki67 (Dako M7240, 1:100) for 30min at room temperature. The staining was visualized by using DakoCytomation EnVision+ System-HRP suitable for rabbit or mouse primary antibodies. Sections stained with polyclonal rabbit/mouse IgG followed by the EnVision+ System was used as negative controls.

### Proteomic analysis on antibody array

The brains from healthy mice and mice injected with MM5 cells were harvested at 9 and 14 days post injection (early growth phase), minced with a scalpel and snap frozen in liquid nitrogen. Half of the frozen tissue from each sample was homogenized and lysed in 1 ml RayBio Cell lysis buffer supplemented with protease inhibitor cocktail (Roche). After centrifugation, the clear supernatants were analyzed on RayBiotech Biotin Label-based Antibody Array 1 (detecting 507 proteins) according to manufacturer instructions followed by image acquisition and data processing as described in Supplementary Information (SI).

## References

[1] Hoek KS, Schlegel NC, Brafford P, Sucker A, Ugurel S, Kumar R, Weber BL, Nathanson KL, Phillips DJ, Herlyn M, Schadendorf D, Dummer R (2006). Metastatic potential of melanomas defined by specific gene expression profiles with no BRAF signature. Pigment Cell Res.

[2] Kulesa PM, Kasemeier-Kulesa JC, Teddy JM, Margaryan NV, Seftor EA, Seftor RE, Hendrix MJ (2006). Reprogramming metastatic melanoma cells to assume a neural crest cell-like phenotype in an embryonic microenvironment. Proc Natl Acad Sci U S A.

[3] Hoek KS, Eichhoff OM, Schlegel NC, Dobbeling U, Kobert N, Schaerer L, Hemmi S, Dummer R (2008). In vivo switching of human melanoma cells between proliferative and invasive states. Cancer Res.

[4] Tap WD, Gong KW, Dering J, Tseng Y, Ginther C, Pauletti G, Glaspy JA, Essner R, Bollag G, Hirth P, Zhang C, Slamon DJ (2010). Pharmacodynamic characterization of the efficacy signals due to selective BRAF inhibition with PLX4032 in malignant melanoma. Neoplasia.

[5] Herlyn M, Thurin J, Balaban G, Bennicelli JL, Herlyn D, Elder DE, Bondi E, Guerry D, Nowell P, Clark WH (1985). Characteristics of cultured human melanocytes isolated from different stages of tumor progression. Cancer Res.

[6] Kusters B, de Waal RM, Wesseling P, Verrijp K, Maass C, Heerschap A, Barentsz JO, Sweep F, Ruiter DJ, Leenders WP (2003). Differential effects of vascular endothelial growth factor A isoforms in a mouse brain metastasis model of human melanoma. Cancer Res.

[7] Xie TX, Huang FJ, Aldape KD, Kang SH, Liu M, Gershenwald JE, Xie K, Sawaya R, Huang S (2006). Activation of stat3 in human melanoma promotes brain metastasis. Cancer Res.

[8] Herrmann JL, Menter DG, Hamada J, Marchetti D, Nakajima M, Nicolson GL (1993). Mediation of NGF-stimulated extracellular matrix invasion by the human melanoma low-affinity p75 neurotrophin receptor: melanoma p75 functions independently of trkA. Mol Biol Cell.

[9] Marchetti D, Denkins Y, Reiland J, Greiter-Wilke A, Galjour J, Murry B, Blust J, Roy M (2003). Brain-metastatic melanoma: a neurotrophic perspective. Pathol Oncol Res.

[10] Rolland Y, Demeule M, Fenart L, Beliveau R (2009). Inhibition of melanoma brain metastasis by targeting melanotransferrin at the cell surface. Pigment Cell Melanoma Res.

[11] Zhang C, Zhang F, Tsan R, Fidler IJ (2009). Transforming growth factor-beta2 is a molecular determinant for site-specific melanoma metastasis in the brain. Cancer Res.

[12] Cruz-Munoz W, Jaramillo ML, Man S, Xu P, Banville M, Collins C, Nantel A, Francia G, Morgan SS, Cranmer LD, O'Connor-McCourt MD, Kerbel RS (2012). Roles for endothelin receptor B and BCL2A1 in spontaneous CNS metastasis of melanoma. Cancer Res.

[13] Lee H, Lin EC, Liu L, Smith JW (2003). Gene expression profiling of tumor xenografts: In vivo analysis of organ-specific metastasis. Int J Cancer.

[14] Lorger M, Felding-Habermann B (2010). Capturing changes in the brain microenvironment during initial steps of breast cancer brain metastasis. Am J Pathol.

[15] Menter DG, Herrmann JL, Nicolson GL (1995). The role of trophic factors and autocrine/paracrine growth factors in brain metastasis. Clin Exp Metastasis.

[16] Nygaard V, Holden M, Loland A, Langaas M, Myklebost O, Hovig E (2005). Limitations of mRNA amplification from small-size cell samples. BMC Genomics.

[17] Shirley SH, Greene VR, Duncan LM, Torres Cabala CA, Grimm EA, Kusewitt DF (2012). Slug expression during melanoma progression. Am J Pathol.

[18] Repovic P, Mi K, Benveniste EN (2003). Oncostatin M enhances the expression of prostaglandin E2 and cyclooxygenase-2 in astrocytes: synergy with interleukin-1beta, tumor necrosis factor-alpha, and bacterial lipopolysaccharide. Glia.

[19] Liu S, Kielian T (2011). MyD88 is pivotal for immune recognition of Citrobacter koseri and astrocyte activation during CNS infection. J Neuroinflammation.

[20] Wilms H, Sievers J, Rickert U, Rostami-Yazdi M, Mrowietz U, Lucius R (2010). Dimethylfumarate inhibits microglial and astrocytic inflammation by suppressing the synthesis of nitric oxide, IL-1beta, TNF-alpha and IL-6 in an in-vitro model of brain inflammation. J Neuroinflammation.

[21] Agrawal D, Chen T, Irby R, Quackenbush J, Chambers AF, Szabo M, Cantor A, Coppola D, Yeatman TJ (2002). Osteopontin identified as lead marker of colon cancer progression, using pooled sample expression profiling. J Natl Cancer Inst.

[22] Shojaei F, Scott N, Kang X, Lappin PB, Fitzgerald AA, Karlicek S, Simmons BH, Wu A, Lee JH, Bergqvist S, Kraynov E (2012). Osteopontin induces growth of metastatic tumors in a preclinical model of non-small lung cancer. J Exp Clin Cancer Res.

[23] Liang Y, Diehn M, Watson N, Bollen AW, Aldape KD, Nicholas MK, Lamborn KR, Berger MS, Botstein D, Brown PO, Israel MA (2005). Gene expression profiling reveals molecularly and clinically distinct subtypes of glioblastoma multiforme. Proc Natl Acad Sci U S A.

[24] Park ES, Kim SJ, Kim SW, Yoon SL, Leem SH, Kim SB, Kim SM, Park YY, Cheong JH, Woo HG, Mills GB, Fidler IJ, Lee JS (2011). Cross-species hybridization of microarrays for studying tumor transcriptome of brain metastasis. Proc Natl Acad Sci U S A.

[25] Pollock PM, Cohen-Solal K, Sood R (2003). Melanoma mouse model implicates metabotropic glutamate signaling in melanocytic neoplasia. Nat Genet.

[26] Wei X, Walia V, Lin JC, Teer JK, Prickett TD, Gartner J, Davis S, Stemke-Hale K, Davies MA, Gershenwald JE, Robinson W, Robinson S, Rosenberg SA, Samuels Y (2011). Exome sequencing identifies GRIN2A as frequently mutated in melanoma. Nat Genet.

[27] Prickett TD, Wei X, Cardenas-Navia I (2011). Exon capture analysis of G protein-coupled receptors identifies activating mutations in GRM3 in melanoma. Nat Genet.

[28] Lin Q, Balasubramanian K, Fan D, Kim SJ, Guo L, Wang H, Bar-Eli M, Aldape KD, Fidler IJ (2011). Reactive astrocytes protect melanoma cells from chemotherapy by sequestering intracellular calcium through gap junction communication channels. Neoplasia.

[29] Liu GJ, Nagarajah R, Banati RB, Bennett MR (2009). Glutamate induces directed chemotaxis of microglia. Eur J Neurosci.

[30] Akbarian S, Huang HS (2009). Epigenetic regulation in human brain-focus on histone lysine methylation. Biol Psychiatry.

[31] Prasmickaite L, Skrbo N, Hoifodt HK, Suo Z, Engebraten O, Gullestad HP, Aamdal S, Fodstad O, Maelandsmo GM (2010). Human malignant melanoma harbours a large fraction of highly clonogenic cells that do not express markers associated with cancer stem cells. Pigment Cell Melanoma Res.

[32] Day CP, Carter J, Bonomi C, Esposito D, Crise B, Ortiz-Conde B, Hollingshead M, Merlino G (2009). Lentivirus-mediated bifunctional cell labeling for in vivo melanoma study. Pigment Cell Melanoma Res.

